# Thyroid hormone levels are linked to history of suicidal behavior in individuals with anxiety and mood disorders

**DOI:** 10.1192/j.eurpsy.2025.722

**Published:** 2025-08-26

**Authors:** V. Jakienė, N. Raškauskienė, A. Podlipskytė, N. Mickuvienė, V. Adomaitienė, E. Zauka, G. Mačys, V. Steiblienė

**Affiliations:** 1 Laboratory of Behavioral Medicine, Neuroscience Institute, Lithuanian University of Health Sciences, Palanga; 2 Clinic of Psychiatry, Lithuanian University of Health Sciences, Kaunas, Lithuania

## Abstract

**Introduction:**

The relation between thyroid function and depression has long been recognized. However, studies analyzing coexistence of thyroid dysfunction and suicidal behavior still offer contradictory results. Thyroid hormones that may play an important role in suicidal behavior have not been thoroughly investigated in the sample of individuals with comorbid anxiety and mood disorders (AMD).

**Objectives:**

The aim of this cross-sectional study was to identify potential associations between thyroid function and suicide attempts (SA) in individuals with AMD.

**Methods:**

This study comprised 147 consecutive participants (mean age 42.4±13.1 years; 69% women). Thirty-five percent (n=51) of the sample was hospitalized after a suicidal attempt, 34 percent (n=50) patients with AMD and without a history of SA, and 31 percent (n=46) individuals without a life time history of mental disorders or suicidal attempt (control group). All participants were interviewed for current psychiatric diagnoses and suicidal behavior using the Mini International Neuropsychiatric Interview. The biochemical blood tests were performed for the concentrations of free thyroxine (FT4), free triiodothyronine (FT3) and thyroid stimulating hormone (TSH). All data were reported as means, standard deviations, or medians (interquartile range [IQR]). Analysis of variance (ANOVA), chi-square test, independent samples t-test, Kruskal-Wallis test, were used for the group comparisons. Multivariable logistic regression analyses were used to assess the associations between thyroid hormones parameters and SA.

**Results:**

All participants had serum FT4, FT3 and TSH level within normal range. Patients with AMD and SA were more likely to be younger than patients with AMD only and the control group (36.1 ± 13.6 vs. 41.5 ± 11.9 and 48.4±12.1 years; p<0.001). There were no significant differences according to gender, BMI, mean values of TSH and FT4 between groups. In our study, FT3, but not the other thyroid axis hormones, was independently associated with suicidal behavior. Patients with AMD and SA had lower FT3 levels in comparison to patients with AMD without SA and the control group (5.20 (0.65) pmol/L vs. 5.52 (0.62) and 5.65 (0.71, p=0.005)).

A multivariable logistic regression revealed that FT3 levels of female SA were significantly lower than non-attempters (4.92 (0.64) pmol/L vs. 5.42 (0.71), p=0.029) and significantly lower than male-attempters (5.61 (0.61), p<0.001). We not found any significant differences with regard to FT3 levels between male SA and non-attempters and controls.

**Conclusions:**

Lower FT3 concentrations, even within the normal reference range, were related to SA in female gender, but not male. This finding supports the importance of evaluating FT3 levels in clinical decision-making on suicide risk of patients with AMD.

**Disclosure of Interest:**

None Declared

